# Adolescent Health Series: The status of adolescent mental health research, practice and policy in sub‐Saharan Africa: A narrative review

**DOI:** 10.1111/tmi.13802

**Published:** 2022-08-17

**Authors:** Miriam Sequeira, Soumya Singh, Luanna Fernandes, Leena Gaikwad, Devika Gupta, Dixon Chibanda, Abhijit Nadkarni

**Affiliations:** ^1^ Addictions Research Group Sangath Goa India; ^2^ Department of Population Health London School of Hygiene and Tropical Medicine London UK; ^3^ Department of Psychiatry, College of Health Sciences University of Zimbabwe Harare Zimbabwe

**Keywords:** adolescent, mental health, sub‐Saharan Africa

## Abstract

Sub‐Saharan Africa (SSA) has the fastest growing adolescent population in the world. In addition to developmental changes, adolescents in SSA face health and socioeconomic challenges that increase their vulnerability to mental ill‐health. This paper is a narrative review of adolescent mental health (AMH) in SSA with a focus on past achievements, current developments, and future directions in the areas of research, practice and policy in the region. We describe the status of AMH in the region, critical factors that negatively impact AMH, and the ways in which research, practice and policy have responded to this need. Depression, anxiety and post‐traumatic stress disorders are the most common mental health problems among adolescents in SSA. Intervention development has largely been focused on HIV/AIDS service delivery in school or community programs by non‐specialist health workers. There is a severe shortage of specialised AMH services, poor integration of services into primary health care, lack of a coordinated inter‐sectoral collaboration, and the absence of clear referral pathways. Policies for the promotion of AMH have been given less attention by policymakers, due to stigma attached to mental health problems, and an insufficient understanding of the link between mental health and social determinants, such as poverty. Given these gaps, traditional healers are the most accessible care available to help‐seeking adolescents. Sustained AMH research with a focus on the socioeconomic benefits of implementing evidence‐based, contextually adapted psychosocial interventions might prove useful in advocating for much needed policies to improve AMH in SSA.

## INTRODUCTION

Adolescence is a period of psychological and physical transformation. Adverse experiences (e.g., childhood neglect or bullying), and changing social environments (e.g., rural to urban migration, exposure to substance using peers, etc.) are some of the risk factors that render the developing adolescent brain and mind vulnerable to mental health problems [1].

In Sub‐Saharan Africa (SSA), adolescents between the ages of 10–19 years constitute 23% (256 million) of the population [2] and are the fastest growing adolescent population in the world, predicted to reach 435 million by 2050 [3]. In addition to developmental changes, adolescents in SSA face several major health and socioeconomic challenges. HIV/AIDS is the leading cause of death among adolescents in Africa [4], and as of 2022, SSA has the highest rate of adolescent pregnancies in the world ‐ at 109 births per thousand adolescent girls aged 10–19 versus the global average of 42 [2]. Approximately every second adolescent in SSA grows up in environments of poverty, with 55% experiencing moderate to severe food insecurity [2]. About 50% of the region has experienced conflict and violence, and this directly affects adolescents as they form a large proportion of those who are killed, raped, or, injured in violent conflicts [5]. Displacement, conflict, and HIV/AIDS impact young people's access to educational opportunities. SSA has the highest rates of education exclusion with more than 33% and 60% of adolescents between the ages of 12–14 years and 15–17 years respectively, being out of school [6]. Rapid urbanisation without the industrial growth required to sustain economic change has led to a rapid increase in urban slums, where many adolescents live in extreme poverty. Little is known about urban adolescent vulnerability to poorer physical and mental health outcomes. Due to weak economic growth and legislation, twice as many young people as adults are unemployed. Evidence from SSA shows that these challenges increase vulnerability to mental health problems among adolescents in this region [7]. Mental ill health in turn may increase chances of adolescents engaging in risky behaviours leading to injuries, HIV/AIDS, sexually transmitted diseases, and teenage pregnancies, thus sustaining this vicious cycle [8, 9].

A recent systematic review estimating the prevalence of mental health problems among adolescents in SSA reported that depression was prevalent in 27% of the population aged 10–19 years, anxiety disorders in 30%, emotional and behavioural problems in 41%, and suicidal ideation in 12%. One study reported a 21% prevalence of post‐traumatic stress disorder (PTSD) [10]. This review did not include prevalence of substance use disorders, which is found to be as high as 42% [11]; but identified a wide variation in prevalence estimates across the sample, depending on their exposure to risk factors. Despite the significant burden, resources and legislation for mental health in the region are inadequate: only 13 of 48 SSA countries have a standalone adolescent mental health (AMH) policy [12]. 40% of African states do not have an allocated budget for mental health, and 24% do not have dedicated mental health legislation [12], resulting in a treatment gap for mental disorders as large as 90% [3]. Despite the high rates of suicides in the region, none of the 18 countries surveyed had a national suicide prevention strategy in place, except for Congo and Madagascar, which were in the process of developing one [13]. It is estimated that, by 2050, all SSA regions will experience a 130% increase in the burden of disease associated with mental disorders [14].

In this context, it is crucial that AMH in the SSA region be prioritised. We have used the social‐ecological framework (SEF) to highlight important developments in AMH research, practice and policy, and suggest future directions. The social‐ecological model of mental health reflects the interplay among macro (societal) and micro (individual) factors [15]. For the purpose of this review, we defined ‘research’ as scientific studies undertaken to understand AMH problems, and address it in any form (e.g., by developing contextually relevant screening and diagnostic tools, intervention development and evaluation). We defined ‘practice’ as existing AMH services in the public health systems, schools or communities–including evidence‐based and traditional healing services. Finally, ‘policy’ is defined as any official statement by a governing body that defines a vision to improve the mental health of a population, with a set of values, principles and objectives, and plan of action. It may or may not include mental health legislation, which are laws protecting the mental health rights of citizens.

We conducted a literature search using the search terms adolescen* OR youth mental health AND intervention* OR treatment OR program* OR technology OR innovations OR polic* OR government polic* OR government program* AND sub‐Sahara Africa OR sub‐Saharan Africa OR each country in the World Bank's list of SSA countries (https://datahelpdesk.worldbank.org/knowledgebase/articles/906519-world-bank-country-and-lending-groups). Data bases searched were Google Scholar, MEDLINE, PsycINFO, Research Gate and Science Direct. Articles published from 2000 onwards were included. Data from relevant studies were extracted into an Excel sheet, and common themes were identified and summarised.

### AMH research and intervention development in SSA

HIV/AIDS and other infectious diseases were the primary focus of adolescent research in SSA, and mental health failed to receive attention, despite being a leading cause of morbidity and mortality in the region [16]. Mental health research in SSA received an impetus from WHO initiatives ‐ the Mental Health Gap Action Program (mhGAP) [17] launched in 2008, and the Comprehensive Mental Health Action Plan 2013–2020 (WHO, 2013), which both aimed to address the treatment gap for mental health problems in low‐resource settings by working with governments, international organisations and other stakeholders to increase the financial and human resource allocation for mental health.

At the individual level, cross‐sectional surveys were conducted to estimate the burden of AMH problems in the region. The Jorns‐Presentati review found AMH prevalence data from 16 different SSA countries, covering 97,616 adolescents from both general and high‐risk populations (poverty, violence, trauma, out of school). 29 screening tools were used, 11 of which were validated to African settings (Jorns‐Presentati et al., 2021). Table 1 lists commonly used screening and diagnostic tools to estimate prevalence of AMH problems in the region.

**TABLE 1:** Adolescent mental health screening and diagnostic tools that have been used in SSA

Tool	Constructs measured	Pros	Cons
Strengths and Difficulties Questionnaire (SDQ) [18]	Conduct problems, hyperactivity/inattention, emotional symptoms, peer problems, and prosocial behaviour	Short, quick administration, measures both mental health difficulties and competencies, and can be administered by a non‐professional with minimal training	Many studies that have used the SDQ in Africa report limited or no validation No contextually established cut‐off scores for SSA Has been used without adhering to developed guidelines in many studies
Patient Health Questionnaire 4‐item version (PHQ‐4) [19] Or the longer PHQ‐9 version	Depression Anxiety	Short and easy to administer. Can be administered by non‐specialist with minimal training. Tested in one study in Tanzania and found to reliably and validly measure core symptoms of depression and anxiety among adolescent girls	Does not measure severity of depression Needs more testing across different adolescent population groups and other SSA countries
10‐item Centre for Epidemiological Studies Depression (CES‐D 10) [20]	Factors indicating depressive symptomatology	Tested with large sample sizes and diverse geographic locations in SSA. Includes data on household‐level socioeconomic indicators	Not tested for construct validity in SSA. Tested only with extremely poor and rural household populations Validation study reports some degree of reporting bias
Beck's Depression Inventory (BDI)‐II (21‐item) [19]	Depression	Short and quick administration (5–10 minutes). Can be used in people ≥13 years Internal consistency established in SSA population. Screening and diagnostic cut off scores for depression established in SSA contexts	Needs validation across different adolescent groups and geographical locations
Major Depression Inventory (MDI) [21]	Depression	Short and quick. Found to be a reliable and valid measure for depression across diverse adolescent groups in Kenya	Although structural validity established, clinical validation to evaluate the most suitable thresholds not conducted
Children's Depression Inventory (CDI) [22]	Depression	Validated for use in African settings. Used in multiple settings across SSA	Since it is a self‐report tool, it can increase likelihood of socially desirable responses

Risk and protective factors specific to adolescents in SSA have been studied at the individual, interpersonal and community levels. Adolescents exposed to violence, conflict, poverty, pregnancy, poor educational opportunities and HIV are at high risk of developing mental health problems, while personal characteristics like personal agency and feeling connected with family, community and animals are resilience enablers. While some socio‐cultural beliefs were found to be resilience enablers, others such as ‘suffering in silence’ were found to be a risk factor for mental health problems among children and adolescents [23].

Interventions to address AMH problems at the individual level were studied within anti‐retroviral treatment facilities, school, family or community settings and typically delivered by non‐specialist providers [24]. Noteworthy studies listed in Table 2 are described below.

**TABLE 2:** Intervention studies to address adolescent mental health care needs in SSA

Program title	Year	Country	Intervention details	Key contribution
Social skills training [25]	2016	Nigeria	8 week, classroom based, teacher delivered intervention to pupils with mild to moderate intellectual disability	Significant improvement in the social skills of pupils
Group cognitive behavioural therapy (CBT) program [26]	2016	Nigeria	5 weekly CBT group sessions delivered by a specialist over 45–60 minutes each	Feasible and effective in reducing depressive symptoms
VUKA family program [27]	2014	South Africa	A 10‐session intervention of approximately 3‐month duration delivered to pre‐adolescents aged 10–13 years and their families.	Improvement in mental health, youth behaviour, HIV treatment knowledge, stigma, communication, and adherence to ART.
The Youth Friendship Bench [28]	2021	Zimbabwe	Culturally contextualised, manualised, peer delivered six‐session problem‐solving therapy to adolescents, 16–19 years of age	Youth reported a positive experience and perceived intervention to offer hope and relief from feelings of isolation and uncertainty, increase manageability of problems, and contribute to feelings of autonomy, resulting in a feeling of optimism about the future
Peer group intervention [29]	2021	South Africa	Peer group clubs designed to build self‐esteem in adolescent girls (aged 15–24), foster supportive peer networks, and provide sexual reproductive health education	Increased self‐esteem, well‐being and perceived social support
Shamiri (thrive) Group intervention [30, 31]	2020	Kenya	School‐based, group intervention delivered once a week by recently graduated students Later developed into a single session digital intervention	Significantly reduced depression and anxiety symptoms and improved social support and academic performance relative to a control group Significantly reduced depressive symptoms
Sauti ya Vijana (The voice of the youth) group based intervention [32]	2020	Tanzania	Group‐based mental health and life skills intervention. 10 group sessions (two sessions held jointly with caregivers) lasting approximately 90 minutes and two individual sessions delivered by trained young adult group leaders who use a manualized protocol that is designed to scale in low‐resource settings	Improved ART adherence and virologic outcomes but no change in mental health outcomes
Integrated approach to addressing the issue of youth depression in Malawi and Tanzania (IACD) [33]	2019	Malawi and Tanzania	A ‘hub and spoke’ model for improving mental health care for young people that included interactive, youth‐informed weekly radio programs, mental health curriculum training for teachers and peer educators in secondary schools, and a clinical competency training program for community‐based health workers	Improved mental health care for young people. Promising guide for adolescent mental health policy development
The African Guide (AG) [34]	2017	Tanzania	The AG is a classroom ready curriculum resource addressing all aspects of mental health literacy. Delivered by teachers via classroom activities	Highly significant improvements in teacher's overall knowledge, including mental health knowledge, and curriculum specific knowledge. Teachers' stigma against mental illness decreased significantly following the training. Students' help‐seeking behaviour increased significantly
Mental health awareness training [35]	2017	Nigeria	School‐based 3‐day mental health training for pupils. Didactic lectures, case history presentations, discussions and role‐play were part of the training.	Significant increase in pupils' knowledge about mental illness and improved attitudes towards help‐seeking for mental health problems.
Families Matter! Program [36]	2016	SSA	Evidence‐based intervention for parents and caregivers of 9–12 year olds to promote positive parenting	Increased parental guidance and support for adolescents living with HIV

The Integrated Approach to Addressing the Challenge of Depression among the youth (IACD) and Shamiri studies are examples of school and community‐based interventions. The IACD in Malawi and Tanzania was successful in enhancing mental health literacy in the community and schools and improving access to effective mental health care for young people with depression. They did so by building the capacity of teachers and community health care providers to identify depression, and by linking schools to community health clinics [33]. Shamiri, a group intervention for adolescent anxiety and depression, was a value‐based multi‐component protocol delivered by non‐specialists in a school setting in Kenya. The intervention showed significant improvements in depression, anxiety and academic grades among the adolescents [30]. The 11 of the 48 SSA countries have developed and tested interventions addressing adolescent trauma (being orphaned, exposed to war or child abuse) [37].

**FIGURE 1:**
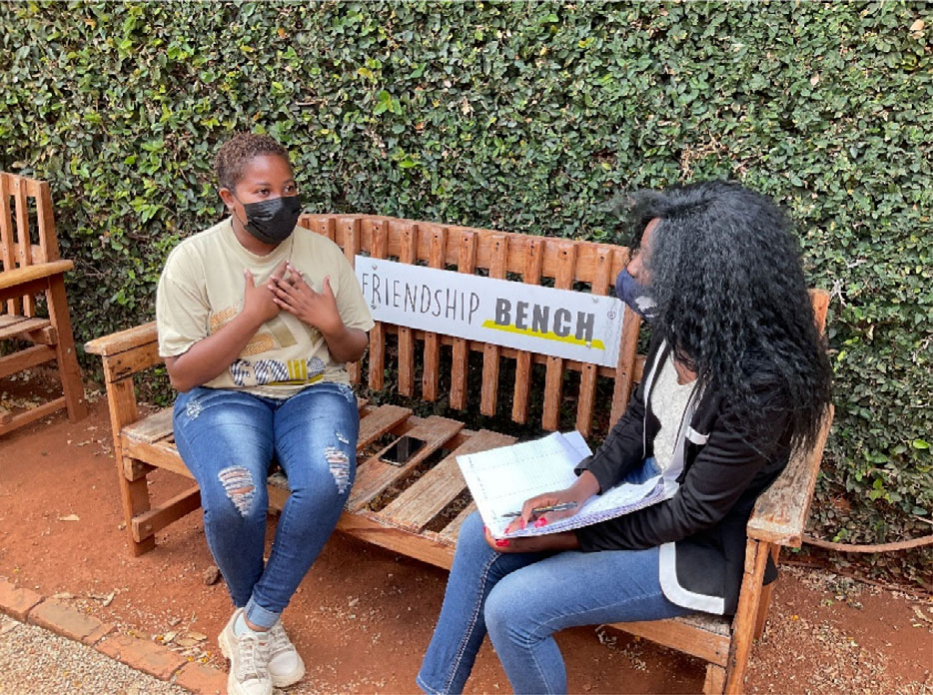
An adolescent speaking to a peer counsellor as part of the Youth Friendship Bench program. The Friendship Bench model, an innovative and evidence based lay counsellor delivered intervention for common mental disorders, has been adapted into the Youth Friendship Bench intervention to address adolescent mental health problems. Sessions are facilitated by youth lay health workers and conducted in community settings like public parks to reach vulnerable populations like pregnant teenagers and juvenile offenders. Participant experiences in this program have been largely positive, perceived to offer hope and relief from negative feelings, increase manageability of problems, and contribute to feelings of autonomy, resulting in a feeling of optimism about the future [28]

More recently, the Youth Friendship Bench (Figure 1) intervention in Zimbabwe, originally a peer‐delivered problem‐solving intervention designed for adults, was adapted for adolescents using [1] delivery by trained students from local communities; [2] delivery in schools and community settings in addition to clinics; [3] special outreach to marginalised youth (pregnant teenagers and young offenders); and [4] community awareness activities to reduce stigma against mental health problems [38].

At the family and community level, the results of intervention programs such as the VUKA family program in South Africa and the Families Matter! Program to promote health and mental health in adolescents living with HIV, delivered to family and community members over weekly sessions to increase guidance and support for adolescents, showed improvements in mental health, HIV treatment literacy, stigma, communication and adherence to medication among participants [27].

The Sauti ya Vijana (The Voice of Youth) group‐based intervention to address youth mental health challenges in Tanzania proved to be successful in promoting resilience by increasing utilisation of new coping skills, improving relationships and self‐confidence, and reducing stigma in youth living with HIV [32]. A peer group intervention for adolescent girls and young women implemented in South Africa successfully promoted self‐esteem, well‐being and perceived social support, which play a protective role in the sexual and reproductive health of this population [29].

Many programs have focused on schools, as these are seen as the most acceptable and feasible environments for AMH interventions. A school‐based mental health training program in Nigeria [35], and school mental health literacy curriculum resource ‘the African Guide’ implemented in Tanzania [34], significantly increased knowledge and attitude scores of students, as well as help‐seeking efficacy. These programs show potential to combat the stigma and discrimination associated with mental health disorders in the region.

More recently, research studies evaluating the impact of unconditional cash transfer programs in South Africa, Tanzania and Malawi found that unconditional cash transfers have stronger benefits for improving mental health, reducing symptoms of depression, and improving education and social support than conditional cash transfer programs [39, 40, 41]. These are promising ways to address the socio‐economic determinants of mental health, and highlight the importance of a multi‐pronged approach to AMH research.

### The status of adolescent mental health practice in SSA

The economic and human resources dedicated to the mental health of children and adolescents remain inadequate in SSA. Situational analyses in the region have highlighted a severe shortage of specialised AMH services, poor integration of services into primary health care, lack of coordinated inter‐sectoral collaboration, and the absence of formal referral pathways [42, 43]. For example, in 2020 there were 0.1 psychiatrists and 0.2 health workers for children and adolescents per 100,000 in the African Region, compared with 9.7 psychiatrists and 12.5 health workers for the same number of children and adolescents in the European region. Speech therapists, occupational therapists and other specialised mental health workers are non‐existent in most of the countries [12]. This shortage is not only because fewer people are trained to be specialists in this region, but also because specialists migrate to higher‐income countries seeking higher salaries, professional development and political stability [44].

At the health systems level, the lack of psychiatric services for children and adolescents is inadequately compensated for by paediatrics and maternal and child health departments, schools, social welfare services and facilities, and juvenile justice system and prisons [45]. Task‐sharing i.e. training non‐specialists to deliver mental health interventions in clinic or community settings, has been the other major development in the region to bridge this care gap. Since the inclusion of mental health in the Millennium Development Goals, the WHO mhGAP framework (which lays out a stepped‐care model for mental health problems) has been used in 23 training courses, 25 clinical practice settings, eight research studies, four contextual adaptations, and two economic analyses in the region [46].

In the absence of formal systems of mental health services for AMH, informal systems, such as traditional and faith healers and non‐governmental organisations have developed and thrived [45]. Traditional healers are mainly herbalists and 'witch doctors,' who use herbal medication or perform rituals and offer counselling to treat their patients. Diagnoses are arrived at after incantations and divinations [47], illnesses are attributed to supernatural imbalances, and plant derivatives are prescribed as treatment.

Almost half of the African population prefers consulting traditional and faith healers for treatment of mental illnesses before accessing formal health care [48]. As mental illnesses are mainly attributed to sorcery or displeased ancestral spirits, patients are directed to receive traditional mental health care [47]. In Nigeria, prayer houses or faith healing centres were the first point of contact for 23%, and traditional healers for 7% of children and adolescents, a majority of which were referred by their relatives, family & friends (91%) [49]. Evidence indicates that traditional healers can effectively deliver psychosocial interventions that reduce mild depressive and anxiety symptoms, but are unable to change the course of severe mental illness [50]. Conversely, accessing these forms of care lead to delayed initiation of formal treatment, and subsequent worsening of symptoms besides other negative effects like poisoning and sometimes death ‐ caused by misidentification of a “medicinal” plant. Additionally, the belief that mental health problems are caused by supernatural powers tend to perpetuate stigma and discrimination [51].

### Adolescent mental health policy in SSA

In an effort to bridge the AMH care gap, some countries in the region have developed adolescent specific mental health policies. With Botswana and Mozambique leading the way in the early 2000s, today 13 of the 48 (13%) SSA countries have a standalone or integrated AMH policy or plan, 37% (18 countries) offer free mental health services to adolescents, and 12% (6 countries) offer mental health services without the need for parental consent [2].

In South Africa, the Integrated School Health Programme, the Primary Health Care Re‐engineering program, and the policy on screening, identification, assessment and support focus on accessible health care services for children and adolescents, and support interventions to enhance learning and eliminate barriers to learning [52, 53, 54]; and the National Mental Health Policy Framework and Strategic Plan 2013–2020 aimed to improve mental health care for all South Africans. None of these policies have a specific focus on the mental health needs of children and adolescents [43].

Nigeria has adopted a national school health policy to ensure the provision of adequate facilities, resources, and programmes for health, safety and security of the school community. It has no guidelines on how to improve the psychosocial environment in schools, provide pre‐entry psychological assessments, or run school counselling services, and hence remains largely unutilized [55]. In other countries such as Ghana, Uganda and Zambia, AMH issues are not included in existing legislation, and incoming or new policies address none, or only few of the six recommendations for the protection of minors from the WHO legislation checklist [56], namely (1) limitation of involuntary placement of minors in mental health facilities, (2) provision of separate living area from adults in mental health facilities, (3) age‐appropriate environment and developmentally appropriate services, (4) adult representation in all matters affecting the minor, (5) consideration of opinions of minors in all issues affecting them, depending on their age and maturity, and (6) banning of all irreversible treatments of children.

### Barriers to AMH research, practice, and policy in SSA

Both demand and supply‐side barriers negatively affect AMH research, practice and policy in the region (Table 3). The major demand‐side barriers exist at the individual, interpersonal and community levels, including poor mental health literacy and social norms that obstruct help‐seeking from evidence‐based interventions, comorbid communicable diseases, household responsibilities, and cultural values that endorse stoicism in the face of adversity.

**TABLE 3:** Barriers to adolescent mental health care in SSA

SEF level	Barriers
Individual	Limited mental health literacy and knowledge about services Explanatory models of mental health problems that might obstruct help‐seeking from evidence‐based medicine Presence of other risk factors like HIV/AIDS, orphanhood, poverty and trauma
Family	Caregivers' level of education Lack of mental health literacy in parents Lack of resources to buy food, drugs or other treatment resources Poverty‐related stress Reliance on traditional healing systems
Community	Stigma around both teenage pregnancy and mental illness Stigma affecting adherence and willingness to seek treatment Community violence/mistreatment Endorsement of traditional healing systems Lack of means of transport to the health facilities War, political conflicts, migration
Health system	Limited capacity to recognise mental distress Negative stereotypes expressed by care providers towards adolescent pregnancy and perinatal depression Overwhelming diseases burden from other diseases and conditions Lack of training to provide mental health interventions Infrastructural barriers to create adolescent friendly services Stock outs, understaffing, limited service availability/and lack of integrated care
Policy	Lack of policy guidance on assessing, monitoring, evaluating, integrating and managing of adolescent mental health problems Lack of mental health policies to encourage help‐seeking Lack or poor implementation of mental health legislation for non‐compliance with policies Lack of policies to retain skilled workers and prevent brain drain to high‐income countries

Supply‐side barriers are health system‐related, and caused by an inadequate understanding of country‐specific estimates of AMH problems due to poor data collection and monitoring within public health systems. Except for a few SSA countries, research regarding prevalence of mental health problems and ensuing adolescent needs, and any assessment of available services is lacking [42]. Heterogeneous methods of screening and diagnosis, lack of contextually validated diagnostic tools (particularly for marginalised sections of society), and different languages have limited the generalisability of findings. There is a need to develop contextually appropriate measurement tools for the region, informed by studies that explore contextual social expectations, experiences, and standards for behaviour in childhood and adolescence [21].

The efforts to improve AMH services in the past were mainly directed towards providing restorative mental health, and had neglected mental health promotion and pre‐emptive social interventions (e.g., antenatal care, interventions to combat malnutrition), thus failing to address the social disadvantages that can lead to mental health challenges [57]. AMH services are largely limited to the province tier of health systems. While efforts have been made to decentralise AMH services, they struggle with task‐sharing, training, and monitoring [58]. The mhGAP recommends a range of pharmacological interventions for low‐resource settings, but many of those drugs are still not available in SSA. Older antipsychotics and tricyclics are easily available; olanzapine and risperidone are available in some settings, while selective serotonin reuptake inhibitors are more difficult to source [42].

Finally, this issue has been given less attention by policy‐makers than other health conditions since stigmatised conditions tend to be given lower priority and there is a lack of understanding about the link between mental health and social determinants such as poverty [56, 59]. Even in countries that have relevant policies, the implementation is insufficiently planned and funded, rendering well‐developed AMH services scarce [60]. Other barriers at this level that affect AMH are political and social instability created by poor governance in many SSA countries, resulting in trauma, destruction of infrastructure, child abuse, poverty and unemployment, which in turn perpetuate AMH problems.

## FUTURE DIRECTIONS

Almost a decade ago Sorsdahl, Stein and Lund [61] called for mental health research in South Africa to focus on the following four areas: [1] Prioritising contextually acceptable mental health intervention development for priority mental health conditions that were identified using contextually validated measurement tools; [2] assessing the feasibility and effectiveness of mental health interventions delivered through task‐sharing; [3] addressing trauma, interpersonal violence and substance use via prevention and management interventions integrated into the public health system; and [4] intersectional research in the areas of substance use, HIV/AIDS and mental health. These are apt for the future of AMH initiatives in SSA even today. Informed by WHO's guidelines on promotive and preventive interventions for AMH [62], we propose the following future directions for the future of AMH sphere in SSA.

### Recommendations for research

We recommend conducting research that examines the equity impact of universally delivered interventions to promote mental health among groups experiencing marginalisation and social exclusion, for example, LGBTI, indigenous populations, and adolescents exposed to violence and/or poverty. Other areas that require attention include needs of at‐risk adolescents such as adolescent fathers, adolescents with disruptive/oppositional behaviours, the impact of social media on AMH, and contextual risk and resilience factors. The evidence base can also be strengthened by evaluation studies ‐ that capture the impact of psychosocial interventions on equity among adolescents living with HIV, and assess the mental health impact of HIV‐related interventions, substance‐use, and suicide and self‐harm prevention interventions.

### Recommendations for practice/implementation

Universally delivered psychosocial interventions to promote positive mental health, prevent mental disorders, self‐harm and suicide, and to reduce risky behaviours are required. These interventions should cover social and emotional learning, and include components such as emotional regulation, problem‐solving, interpersonal skills, mindfulness, assertiveness and stress management. Universal interventions in schools or delivered through mobile phones may be easier to scale up as they can reach a wider population, and are less likely to cause stigmatisation.

Interventions for at‐risk adolescents that include stress management and relaxation strategies have been effective. Trauma‐focused cognitive behavioural therapy (CBT) has shown positive effects on reducing symptoms of depression, anxiety and stress; and group‐based CBT interventions have been effective for adolescents exposed to stressful events. Interventions that built cognitive and behavioural skills have been effective in in promoting mental functioning and mental well‐being, and improve school attendance among pregnant adolescents and young mothers. These interventions could be delivered through digital, school or community‐based task‐sharing models (including traditional healers), and made more accessible by integrating into the public health system. When working with marginalised populations, it is important to take into account age and gender‐specific vulnerabilities and socio‐cultural risk and resilience factors.

### Policy recommendations

Without national action to enhance budgetary allocation for mental health, design monitoring and evaluation protocols, and improved accountability through mental health legislation, the recommendations for research and practice are in vain. Considering the effectiveness of cash disbursements on mental health, particularly the reduction in suicide risk, these could be included as a priority intervention in mental health policies.

Funding support for initiatives like the African Mental Health Research Initiative [63], a consortium of four African universities and SUCCEED Africa, help build regional leadership and capacity for innovations in AMH research, practice and policy [64]. An example of one such network is the Africa Research, Implementation Science and Education (ARISE) Network whose Adolescent Health Study collected community‐level data from nine countries in SSA and published 12 research papers [65].

## CONCLUSION

AMH has been neglected as a leading cause of morbidity and mortality in SSA. Research to understand AMH issues in more detail is required, as are networks of skilled African mental health researchers. Demonstrating the benefits of evidence‐based, contextually‐adapted psychosocial interventions might prove useful in advocating for much needed policy commitments.
